# Prevalence, progress, and subgroup disparities in pharmacological antidepressant treatment of those who screen positive for depressive symptoms: A repetitive cross-sectional study in 19 European countries

**DOI:** 10.1016/j.lanepe.2022.100368

**Published:** 2022-03-28

**Authors:** Shanquan Chen, Tamsin J. Ford, Peter B. Jones, Rudolf N. Cardinal

**Affiliations:** a Department of Psychiatry, University of Cambridge, Cambridge CB2 0SZ, United Kingdom; b Cambridgeshire and Peterborough NHS Foundation Trust, CB21 5EF, United Kingdom; c NIHR Applied Research Collaboration, East of England, United Kingdom

**Keywords:** Depression, Antidepressants, Disparity, European, ADL, activities of daily living, AOR, adjusted odds ratio, ARC, applied research collaboration, BNF, British national formulary, CI, confidence interval, EMHAP, European mental health action plan, GDP, gross domestic product, GHQ-12, 12-item general health questionnaire, GNI, gross national income per capita, GP, general practitioner, HSE, health survey for England, IAPT, improving access to psychological therapies, IMD, index of multiple deprivation, NHS, National Health Service, NIHR, National Institute for Health Research, OR, odds ratio, SHARE, survey of health, ageing and retirement in Europe, STROBE, strengthening the reporting of observational studies in epidemiology, UK, United Kingdom, USA, United States of America, WHO, World Health Organization

## Abstract

**Background:**

The European Mental Health Action Plan (EMHAP) 2013–2020 promoted community-based mental health services. One potential success indicator is the provision of antidepressant medication to those needing it.

**Methods:**

Public data from two surveys (Health Survey for England, UK; Survey of Health, Ageing and Retirement in Europe) covered 19 European countries across EMHAP phases one (2011–2015) and two (2015–2018). People screening positive for depressive symptoms by self-report were included. The primary outcome was antidepressant use: using country-specific weighted regression models, we estimated temporal trends and subgroup disparities in antidepressant receipt, with secondary analysis by country-level measures including healthcare expenditure.

**Findings:**

Across 37,250 participants, after controlling for age, sex, wealth, and physical disability, antidepressant use (amongst those screening positive) increased significantly in 14/19 countries, with the smallest increase being in Slovenia (adjusted OR[AOR] for trend=1.68[1.20–2.36]) and the highest increase being in Germany (AOR for trend=10.07[7.54–13.46]) and Austria (AOR for trend=10.07[7.32–13.74]). The overall proportion using antidepressants was positively associated with national health expenditure (coefficient=5.43[1.62–9.25]), but not with gross national income per capita or the number of psychiatrists, general practitioners, or psychiatric hospital beds. In 15/19 countries, antidepressants were used less by ≥65-year-olds than 50–64-year-olds, with the smallest differential reported in Luxembourg (AOR=0.70[0.49, 0.98]) and the highest in Germany (AOR=0.28[0.21, 0.37]); this disparity widened in 12/15 countries. Men used antidepressants less than women in 8/19 countries, across phases. In 13/19 countries, people with physical disability were more likely to receive antidepressants, with the smallest gap in Italy (AOR=1.42[1.12–1.80]) and the largest in Israel (AOR=2.34[1.46–3.74]); this disparity narrowed in 5/13 countries. Disparity by wealth was found in 8/19 countries, but its temporal trend varied.

**Interpretation:**

Usage of antidepressants by those with depressive symptoms has increased, with wide variation between countries and subgroups. Disparities across age, sex, and disability should prompt further research.

**Funding:**

Medical Research Council (grants MC_PC_17213 and MR/W014386/1), UK National Institute for Health Research (NIHR) Applied Research Collaboration (ARC) East of England, NIHR Cambridge Biomedical Research Centre (BRC-1215-20014).

**Research in context**

**Evidence before this study**

We conducted a literature search in PubMed and Web of Science for papers published before 10 September 2021, using the terms ‘(“depression” OR “depressive”) AND (“antidepressant” OR “medicine” OR “drug”)’. The search terms were restricted to title and abstract. There were no language restrictions. Mental disorders are one of the top public health challenges in the World Health Organization (WHO) European Region, affecting about 25% of the population in a lifetime. Concerted efforts were adopted by European countries to enable community-based mental health services to be accessible to all groups in the population. Some studies have evaluated antidepressant usage among European countries before the European Mental Health Action Plan (EMHAP, 2013–2020), finding an increasing trend during 2007–2011, with women and the elderly having the highest usage of antidepressants. In addition, between-country variability in antidepressant consumption was found to be correlated with pharmaceutical expenditure, number of general practitioners, healthcare spending, and public attitudes towards mental illness. 2020 marked the end of the EMHAP, but no subsequent study has examined changes in treatment and what is still to be achieved.

**Added value of this study**

This is the first study to assess usage of antidepressants by those who screen positive for depressive symptoms following the end of the EMHAP. Using repetitive cross-sectional population-based datasets covering 19 countries (with participants aged 50+ for 18 countries and 13+ for one country), we found significant increases in usage of antidepressants by people screening positive for depressive symptoms. Among those screening positive, the mean percentage receiving antidepressants increased from 24.5% to 38.7% from the first phase (2011–2015) to the second (2015–2018). There was wide variation between countries, with the lowest prescription rate in Estonia (13.5%) and highest prescription rate in Austria (81.3%) during the second phase. Salient subgroup disparities were found for sex, age (≥65 versus 50–64), and physical disability. Across phases, the age disparity widened, the sex disparity persisted, and the physical disability disparity narrowed. Disparity by wealth status was inconsistent and variable. We also examined antidepressant receipt in relation to five country-level measures of affordability and availability of resources, and found that the percentage receiving antidepressants was positively associated with national health expenditure, but not with measures of affordability/availability of resources. An increase in antidepressant usage was associated with a decrease in psychiatric inpatient beds, but not with changes in four other country-level factors.

**Implications of all the available evidence**

Our findings suggest that characteristics other than clinical need influence access to, or usage of, antidepressants for those who screen positive for depressive symptoms. Non-pharmacological treatments of depression are also available, and this may represent an important factor in determining the observed differences in antidepressant use. Commissioners, practitioners, and policy makers could use our findings as one starting point to investigate and improve appropriate access to mental health treatments in their regions.

Alt-text: Unlabelled box

## Introduction

Mental disorders are one of the top public health challenges in the World Health Organization (WHO) European Region, affecting about 25% of the population in a lifetime.^1^ One of the aims of the WHO's European Mental Health Action Plan (EMHAP) 2013–2020 was to enable community-based mental health services to be accessible to all groups in the population.^1^ Following the end of this plan, timely evaluation is required to measure changes and establish where action is still needed.

Access to pharmacological treatment for mental disorder is a significant part of community-based mental health services.^2^ For instance, antidepressants are effective in around 60–70% of individuals with moderate to severe depression, and can be prescribed by non-specialist health professionals (e.g. general practitioners) with training.^3^ Psychopharmacological medicines are part of the WHO List of Essential Medicines; their availability and accessibility is a core mental health indicator for a health system.^3^

Some studies had evaluated antidepressant usage among European countries before the EMHAP, finding an increasing trend during 2007–2011, with women and the elderly having the highest levels of antidepressant use.^4^^,^^5^ In addition, between-country variability in antidepressant consumption was found to be correlated with pharmaceutical expenditure, number of general practitioners, healthcare spending, and public attitudes towards mental illness.^4^^,^^5^ However, to our knowledge, no corresponding study has followed the completion of the EMHAP.

Our primary aim was to evaluate temporal trends in pharmacological treatment of individuals who screened positive for depressive symptoms in 19 European countries (a subset of member states of the WHO Regional Office for Europe) after the EMHAP, by age, sex, wealth status, and physical disability. The second aim was to examine the percentage receiving antidepressants in relation to five country-level measures of affordability and availability of healthcare resources.

## Methods

### Study design and participants

We used publicly available data from two surveys: the Health Survey for England (HSE) in the UK,^6^ and the Survey of Health, Ageing and Retirement in Europe (SHARE) for another 18 countries.^7^ In brief, the HSE is an annual population-based survey of people aged 13 or over in England, UK, which uses stratified multistage probability sampling to produce nationally representative estimates of the English population. SHARE is a biennial multi-nationally representative individual survey of people aged 50 or over, with centrally standardized methods across its participating countries for the explicit purpose of cross-country comparison. SHARE participants are sampled based on probability selection methods; sample frames (mostly population registers) are chosen in accordance with the best available frame resources in the country to achieve full probability sampling, though there are small variations in sampling frames.^7^^,^^8^ In both surveys, participants were interviewed by trained personnel using computer-assisted interviewing.^6^^,^^7^ Items included sociodemographic characteristics (age, sex, and wealth status), activities of daily living (ADL), a measure of depressive symptoms, and use of antidepressants. Differences between HSE and SHARE include: (1) a single country within a study (HSE) versus multiple countries (SHARE); (2) age range: HSE includes both adults (16+) and children (0–15) but only children aged 13+ are interviewed directly,^9^ while the age range is 50+ in SHARE^10^; (3) a sample of people in private residential addresses (HSE) versus people in private residences ± people living in institutions (SHARE, varying by country); (4) repeated resampling with facilities for further longitudinal linkage (HSE) versus longitudinal re-interviewing plus sample refreshment (SHARE); (5) interviewer visit then nurse visit (HSE) versus interviewer only (SHARE); (6) the measure of depressive symptoms used (see below); (7) whether medication usage was recorded by a nurse (HSE) or self-recalled by participants (SHARE) (see below); (8) the wealth measure (see below).^11^^,^^12^ Detailed descriptions of HSE and SHARE, including the sampling methods, quality control procedures, and data collection, can be found elsewhere.^10^^,^^11^^,^^13^^,^^14^

The data collected by SHARE in each wave that was required for our analysis did not cover all countries and ages. We included the available data nearest to the relevant implementation times of the EMHAP. We excluded data collected by SHARE in 2020, in view of the unusual influence that the COVID-19 pandemic is likely to have had on both services and data collection.^15^^,^^16^ We retrieved data from HSE and earlier versions of SHARE, covering 19 European countries, with a first (or start) phase of 2011–2015 and a second (or end) phase of 2015–2018.

Our analysis only included participants scoring above established clinical cut-offs for depressive symptoms. The instrument used by HSE was the 12-item General Health Questionnaire (GHQ-12), which rates concentration, sleep loss, sense of contribution, decision-making capability, strain, overcoming difficulties, enjoyment/anhedonia, problem-facing, low mood, loss of confidence, worthlessness, and happiness,^17^ via 12 questions each scored 0–1, with a cut-off point for ‘caseness’ of 4/12.^6^ The instrument used by SHARE was the EURO-D scale, validated to measure depressive symptoms; this consists of 12 dichotomous items indicating the presence or absence of depressed mood, pessimism, death wish, guilt, irritability, tearfulness, fatigue, sleep disturbance, loss of interest, loss of appetite, reduced ability to concentrate, and loss of capacity to enjoy things over the preceding month, with a screening cut-off point of 4/12.^7^ We took these thresholds as reflecting screening positive for depressive symptoms, accepting the caveats and limitations that self-report scales entail, with both false positives and false negatives with respect to a diagnosis of depression.^18^

Further details, including a flowchart of population selection and lists of the countries included and the years covered in this study, are provided in the **Supplementary Materials**.

The data are publicly available. The use of secondary de-identified data made this study exempt from institutional review board review. Participants in the original studies gave informed consent and each study was approved by a relevant ethics body: for HSE, the London Medical Research Ethics Council and/or local Research Ethics Councils prior to each annual data collection cycle^6^^,^^9^; for SHARE, the Ethics Council of the Max Planck Society plus ethics committees in participating countries.^19^

### Outcomes of interest

*Utilization of antidepressants (yes vs no),* noted by a nurse in HSE based on the participants' prescription records and the medications they were taking,^6^ or self-recalled by participants in SHARE.^8^ In HSE, participants were asked if they were taking any medications prescribed for them by a doctor or nurse; if so, they were asked to show the medications to the assessing nurse, who classified them according to British National Formulary (BNF) sections^20^; the definition of “antidepressant medications” included tricyclic and related antidepressant drugs, monoamine oxidase inhibitors, selective serotonin re-uptake inhibitors, and other antidepressant drugs (BNF section 4.3, “antidepressant drugs”).^21^ In SHARE, participants were asked to indicate whether they were taking “drugs for anxiety or depression”, “at least once a week”.^8^

### Other variables

#### Wealth status (categorical variable with five levels)

The wealth measure in HSE was the 2015 UK Index of Multiple Deprivation (IMD). The IMD, which is calculated for a small geographical area of residence (a national census Lower Layer Super Output Area, mean population 1500), is the official measure of relative deprivation in England, and incorporates seven domains: income, employment, health and disability, education, barriers to housing and services, living environment and education, and crime.^22^ (A potential weakness of IMD is that individual household income may differ from the mean level of wealth/deprivation associated with this small geographical area; a potential strength is its multi-domain nature. HSE collects direct household income directly but the data are provided in categorical format prohibiting the calculation of quintiles.) The wealth measure in SHARE was self-reported gross total household income. Both were divided into five quintiles for analysis, with quintiles calculated within each country and across all survey participants (including those not screening positive for depressive symptoms); we took the lowest quintile as the reference category.

#### Physical disability (yes vs no)

Disability was assessed by six basic ADLs (such as getting out of bed and walking across a room) and nine “instrumental” ADLs (such as shopping for groceries and preparing a hot meal).^23^ Participants who responded positively to one or more items (indicating difficulty) were defined as having a physical disability.^23^

#### Country-level measures

We extracted data on five measures of affordability and availability of resources for services from Eurostat, the statistical office run by the European Commission and the official provider of statistics at European level.^24^ These were (1) gross national income per capita (GNI, in US$1000), (2) public expenditure on health as a percentage of GDP, (3) psychiatrists per 100,000 inhabitants, (4) general practitioners (GPs) per 100,000 inhabitants, and (5) psychiatric care beds in hospitals per 100,000 inhabitants (being, along with the number of psychiatrists, a potential indirect measure of resources in community psychiatric care).

### Statistical analysis

Data were analysed for each country separately. This makes within-country comparisons (analyses of changes over time) robust to any between-country or between-survey differences between survey methods (as summarized above), since survey methods were consistent for any given country over time. Repeated cross-sectional sampling is a standard method for measuring changes,^25^^,^^26^ including for the assessment of trends relating to depression based on screening tools.^27^ Survey weighting was used to adjust for the complex survey design, including the unequal probability of selection, clustering, and stratification, to make estimates representative of each country. The weight values were provided directly in the HSE and SHARE datasets. Details of how the weights were calculated can be found elsewhere.^13^^,^^14^

To estimate temporal trends, we fitted country-specific weighted logistic regression models (one model per country), with antidepressant receipt as the dependent variable and phase (start phase [reference] vs. end phase) as the predictor, whilst controlling for age, sex, wealth status, and disability. To estimate subgroup disparity, we added the interaction term between the relevant subgroup variable and phase.

We explored further the association of the percentage receiving antidepressants with five country-level factors relating to the affordability and availability of health care resources. We used linear regression with the percentage receiving antidepressants as the outcome and these country-level factors as predictors. We also explored associations between the change in antidepressant use and changes in these measures during the period studied. If all countries used different methods, comparison of absolute values across countries would be impossible and comparison of changes would require the assumption that methodological differences did not affect rates of change across countries. However, cross-country comparison is fully supported by the standardized methods used by SHARE, though there are caveats with regard to HSE/SHARE (UK/other country) cross-comparison (discussed in detail later). Cross-country comparison using SHARE data is an established technique and several studies have addressed other cross-country questions in the domain of depressive symptoms using this data source.^7^^,^^28^^,^^29^

The data are complete except for the wealth variable, which had 615 (1.7%) records with missing values. For wealth, we imputed data by using multiple imputations with chained equations and generated five imputed data sets to reduce bias and maintain power.^30^

We used R version 3.6.0. We report two-tailed P values and 95% confidence intervals (CIs) throughout. P<.05 was considered statistically significant. Results are reported following the STROBE checklist for cohort studies.

### Role of the funding source

The funder of the study had no role in study design, data collection, data analysis, data interpretation, or writing of the article. The views expressed are those of the authors and not necessarily those of the NHS and the NIHR.

## Results

37,250 participants from 19 countries, who all screened positive for depressive symptoms, were included in this analysis (23,213 participants in the start phase, 2011–2015, and 14,037 participants in the end phase, 2015–2018). Table 1 shows demographics by country and study phase. Among these participants, 68.4% were female and 38.2% had a physical disability. People aged 65 or over accounted for 59.1%, followed by people aged 50–64 (36.7%). With respect to the wealth measure, 16.2% (start phase) or 15.2% (end phase) were in the most affluent quintile (across all survey participants including those not screening positive for depressive symptoms), whilst 22.6% were in the least affluent quintile (Table 1), evidence of a significantly higher prevalence of depressive symptoms amongst the less wealthy (start phase: *χ*^2^_4_ = 653.07, *p* < 2.2 × 10^−16^; end phase: *χ*^2^_4_ = 509.61, *p* < 2.2 × 10^−16^).

**Table 1:** Demographic characteristics of included participants, who all screened positive for depressive symptoms, by country and study phase.

Country	Data period	Phase	*N*	Age, *n* (%)					Sex (= female)	Wealth status quintile, *n* (%)					Physical disability (= yes)
				13–19	20–24	25–49	50–64	65+	*n* (%)	1 (Low)	2	3	4	5 (High)	*n* (%)
Austria	2013–2017	start	936	–	–	–	346 (37)	590 (63)	657 (70.2)	246 (26.3)	208 (22.2)	181 (19.3)	152 (16.2)	149 (15.9)	409 (43.7)
		end	337	–	–	–	89 (26.4)	248 (73.6)	239 (70.9)	95 (28.2)	66 (19.6)	76 (22.6)	53 (15.7)	47 (13.9)	174 (51.6)
Belgium	2013–2017	start	1832	–	–	–	911 (49.7)	921 (50.3)	1222 (66.7)	444 (24.2)	380 (20.7)	376 (20.5)	342 (18.7)	290 (15.8)	766 (41.8)
		end	915	–	–	–	327 (35.7)	588 (64.3)	653 (71.4)	249 (27.2)	230 (25.1)	151 (16.5)	155 (16.9)	130 (14.2)	426 (46.6)
Czech Republic	2013–2017	start	1557	–	–	–	637 (40.9)	920 (59.1)	1119 (71.9)	273 (17.5)	457 (29.4)	319 (20.5)	248 (15.9)	260 (16.7)	641 (41.2)
		end	487	–	–	–	107 (22)	380 (78)	374 (76.8)	52 (10.7)	189 (38.8)	109 (22.4)	76 (15.6)	61 (12.5)	254 (52.2)
Denmark	2013–2017	start	863	–	–	–	466 (54)	397 (46)	577 (66.9)	224 (26)	181 (21)	175 (20.3)	167 (19.4)	116 (13.4)	297 (34.4)
		end	358	–	–	–	133 (37.2)	225 (62.8)	252 (70.4)	98 (27.4)	70 (19.6)	89 (24.9)	48 (13.4)	53 (14.8)	161 (45)
Estonia	2013–2015	start	2073	–	–	–	682 (32.9)	1391 (67.1)	1430 (69)	361 (17.4)	655 (31.6)	487 (23.5)	273 (13.2)	297 (14.3)	943 (45.5)
		end	1907	–	–	–	654 (34.3)	1253 (65.7)	1347 (70.6)	329 (17.3)	601 (31.5)	444 (23.3)	292 (15.3)	241 (12.6)	828 (43.4)
France	2013–2017	start	1659	–	–	–	673 (40.6)	986 (59.4)	1146 (69.1)	404 (24.4)	416 (25.1)	306 (18.4)	273 (16.5)	260 (15.7)	604 (36.4)
		end	623	–	–	–	165 (26.5)	458 (73.5)	457 (73.4)	178 (28.6)	117 (18.8)	124 (19.9)	101 (16.2)	103 (16.5)	261 (41.9)
Germany	2013–2017	start	1498	–	–	–	834 (55.7)	664 (44.3)	984 (65.7)	365 (24.4)	368 (24.6)	240 (16)	265 (17.7)	260 (17.4)	492 (32.8)
		end	387	–	–	–	155 (40.1)	232 (59.9)	251 (64.9)	108 (27.9)	87 (22.5)	52 (13.4)	77 (19.9)	63 (16.3)	153 (39.5)
Greece	2015–2017	start	1566	–	–	–	631 (40.3)	933 (59.7)	1067 (68.1)	337 (21.5)	354 (22.6)	286 (18.3)	278 (17.8)	311 (19.9)	601 (38.4)
		end	528	–	–	–	83 (15.7)	445 (84.3)	383 (72.5)	103 (19.5)	181 (34.3)	99 (18.8)	79 (15)	66 (12.5)	286 (54.2)
Israel	2013–2015	start	646	–	–	–	180 (27.9)	466 (72.1)	415 (64.2)	182 (28.2)	169 (26.2)	116 (18)	97 (15)	82 (12.7)	369 (57.1)
		end	584	–	–	–	136 (23.3)	448 (76.7)	385 (65.9)	185 (31.7)	131 (22.4)	116 (19.9)	77 (13.2)	75 (12.8)	301 (51.5)
Italy	2013–2017	start	1732	–	–	–	615 (35.5)	1116 (64.5)	1193 (68.9)	402 (23.2)	433 (25)	351 (20.3)	294 (17)	252 (14.5)	623 (36)
		end	784	–	–	–	147 (18.8)	637 (81.2)	531 (67.7)	209 (26.7)	190 (24.2)	147 (18.8)	109 (13.9)	129 (16.5)	330 (42.1)
Luxembourg	2013–2015	start	489	–	–	–	274 (56)	215 (44)	307 (62.8)	108 (22.1)	127 (26)	96 (19.6)	80 (16.4)	78 (16)	172 (35.2)
		end	489	–	–	–	255 (52.1)	234 (47.9)	329 (67.3)	114 (23.3)	105 (21.5)	89 (18.2)	90 (18.4)	91 (18.6)	142 (29)
Netherlands	2013–2017	start	832	–	–	–	418 (50.2)	414 (49.8)	553 (66.5)	209 (25.1)	199 (23.9)	129 (15.5)	154 (18.5)	141 (16.9)	289 (34.7)
		end	893	–	–	–	386 (43.2)	507 (56.8)	584 (65.4)	208 (23.3)	195 (21.8)	176 (19.7)	168 (18.8)	146 (16.3)	346 (38.7)
Poland	2015–2017	start	724	–	–	–	289 (39.9)	435 (60.1)	479 (66.2)	124 (17.1)	235 (32.5)	133 (18.4)	95 (13.1)	137 (18.9)	284 (39.2)
		end	691	–	–	–	201 (29.1)	490 (70.9)	470 (68)	116 (16.8)	197 (28.5)	149 (21.6)	117 (16.9)	112 (16.2)	302 (43.7)
Portugal	2011–2015	start	863	–	–	–	413 (47.9)	450 (52.1)	605 (70.1)	209 (24.2)	197 (22.8)	145 (16.8)	158 (18.3)	154 (17.8)	343 (39.7)
		end	758	–	–	–	286 (37.7)	472 (62.3)	528 (69.7)	160 (21.1)	185 (24.4)	159 (21)	132 (17.4)	122 (16.1)	337 (44.5)
Slovenia	2013–2015	start	750	–	–	–	280 (37.3)	470 (62.7)	531 (70.8)	135 (18)	213 (28.4)	158 (21.1)	140 (18.7)	104 (13.9)	254 (33.9)
		end	1077	–	–	–	403 (37.4)	674 (62.6)	736 (68.3)	239 (22.2)	245 (22.7)	248 (23)	145 (13.5)	200 (18.6)	426 (39.6)
Spain	2013–2017	start	2089	–	–	–	698 (33.4)	1391 (66.6)	1436 (68.7)	428 (20.5)	559 (26.8)	414 (19.8)	344 (16.5)	344 (16.5)	936 (44.8)
		end	945	–	–	–	180 (19)	765 (81)	685 (72.5)	168 (17.8)	268 (28.4)	214 (22.6)	141 (14.9)	154 (16.3)	472 (49.9)
Sweden	2013–2017	start	982	–	–	–	374 (38.1)	608 (61.9)	669 (68.1)	266 (27.1)	208 (21.2)	202 (20.6)	137 (14)	169 (17.2)	284 (28.9)
		end	371	–	–	–	61 (16.4)	310 (83.6)	264 (71.2)	117 (31.5)	77 (20.8)	66 (17.8)	67 (18.1)	44 (11.9)	126 (34)
Switzerland	2013–2017	start	609	–	–	–	269 (44.2)	340 (55.8)	417 (68.5)	134 (22)	132 (21.7)	131 (21.5)	106 (17.4)	106 (17.4)	175 (28.7)
		end	238	–	–	–	64 (26.9)	174 (73.1)	161 (67.6)	55 (23.1)	40 (16.8)	40 (16.8)	48 (20.2)	55 (23.1)	87 (36.6)
UK	2014–2018	start	1513	89 (5.9)	60 (4)	586 (38.7)	409 (27)	369 (24.4)	993 (65.6)	389 (25.7)	296 (19.6)	298 (19.7)	278 (18.4)	252 (16.7)	162 (10.7)
		end	1665	106 (6.4)	90 (5.4)	600 (36)	457 (27.4)	412 (24.7)	1066 (64)	383 (23)	386 (23.2)	346 (20.8)	307 (18.4)	243 (14.6)	161 (9.7)
Total		start	23213	89 (0.4)	60 (0.3)	586 (2.5)	9399 (40.5)	13076 (56.3)	15800 (68.1)	5240 (22.6)	5787 (24.9)	4543 (19.6)	3881 (16.7)	3762 (16.2)	8644 (37.2)
		end	14037	106 (0.8)	90 (0.6)	600 (4.3)	4289 (30.6)	8952 (63.8)	9695 (69.1)	3166 (22.6)	3560 (25.4)	2894 (20.6)	2282 (16.3)	2135 (15.2)	5573 (39.7)

Categorical variables are reported as number (percentage). Wealth quintiles are calculated with respect to all survey participants (including those not screening positive for depressive symptoms). “–” means not applicable.

**Figure 1:**
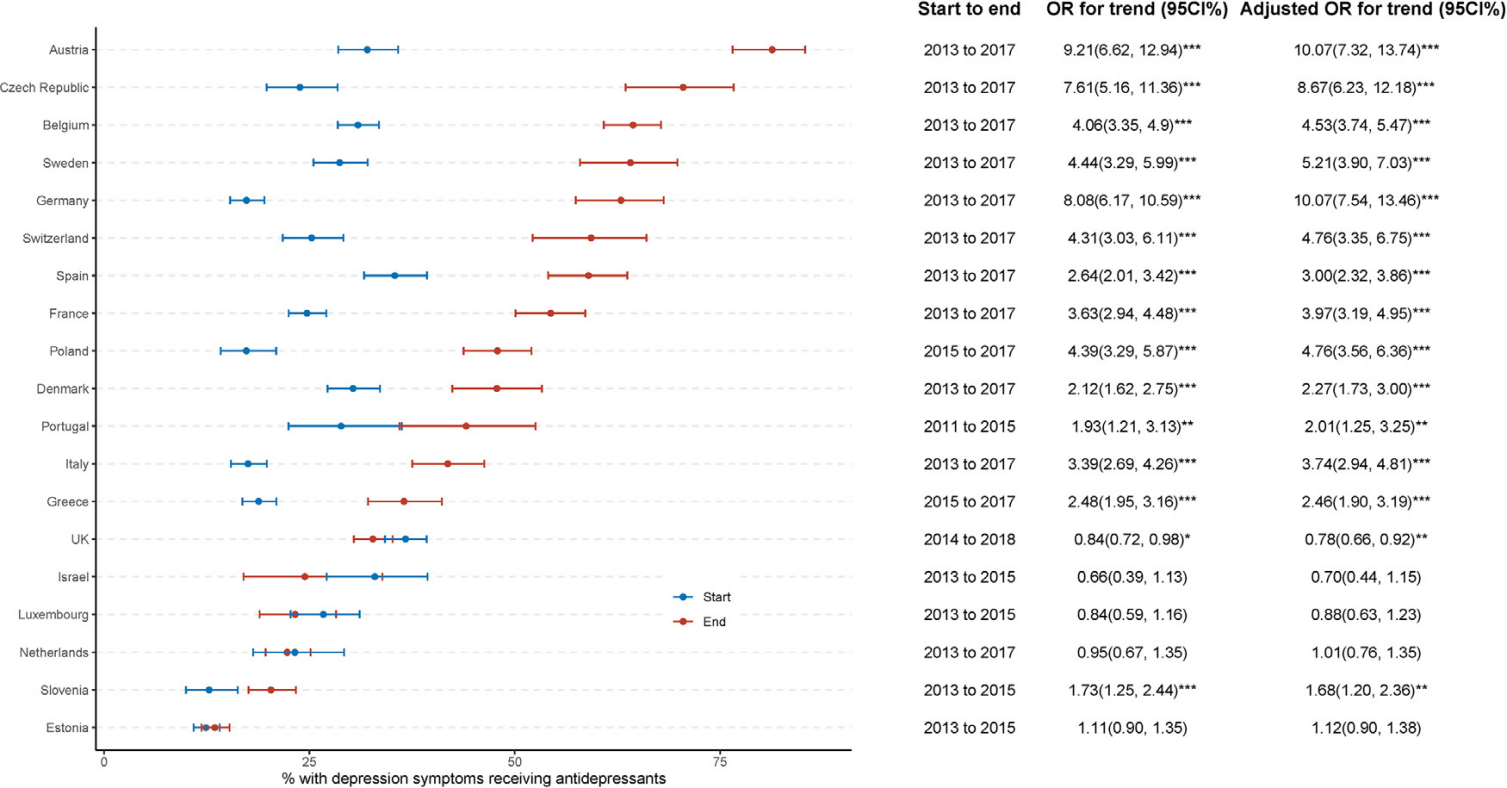
**Percentage receiving antidepressants (among those screening positive for depression) by phase.** Odds ratios (ORs) and their 95% confidence intervals (CIs) were estimated from weighted logistic regression, with antidepressant receipt as the dependent variable and phase (end, vs start [reference]) as the predictor. Adjusted ORs (with 95%CIs) were estimated from the model controlling for age, sex, wealth status, and disability. OR >1 indicates a higher likelihood of receiving antidepressants during the end phase compare to the start phase. *** *p* < 0.001; ** *p* < 0.01; * *p* < 0.05; . *p* < 0.1.

Among people who screened positive for depressive symptoms, the percentage receiving antidepressants varied substantially between countries, with the lowest prescription rate being in Estonia (13.5%, 95%CI [11.9%, 15.3%]) and the highest prescription rate being in Austria (81.3% [76.5%, 85.4%]) during 2015–2018 (Figure 1). After controlling for age, sex, wealth, and disability, there was a statistically significant increase in the proportion receiving antidepressants in 14 of 19 countries, with the smallest increase being in Slovenia (AOR for trend 1.68 [1.20, 2.36]) and the highest increase being in Germany (AOR for trend 10.07 [7.54, 13.46]) and Austria (AOR for trend 10.07 [7.32, 13.74]) (Figure 1). The percentage receiving antidepressants decreased in the UK (AOR for trend 0.78 [0.66, 0.92]), and did not change significantly in Israel, Luxembourg, the Netherlands, or Estonia (Figure 1).

Table 2 shows that an increase in public expenditure on health of 1% of GDP was significantly associated with a 5.43 [1.62, 9.25] per cent increase in antidepressant receipt, while the percentage receiving antidepressants was not associated with the other four country-level factors. Table 2 also shows that change (across phases) in the percentage receiving antidepressants was negatively and significantly associated with the change in the number of psychiatric beds (coefficient −2.22 [−4.34, −0.11]) but not with the other four country-level factors.

**Table 2:** Associations between antidepressant receipt (among those screening positive for depression) and national-level health system factors.

National-level variables	Association with percentage receiving antidepressants		Association with change in the percentage receiving antidepressants	
	Coefficient (95% CI)	*p*	Coefficient (95% CI)	*p*
Psychiatrists per 100 000 inhabitants	−1.27 (−2.67, 0.12)	0.072	0.97 (−7.18, 9.12)	0.7997
General practitioners per 100 000 inhabitants	0.05 (−0.18, 0.28)	0.657	0.20 (−0.89, 1.30)	0.6933
Gross national income per capita (1000 US dollars)	0.26 (−0.11, 0.64)	0.165	3.60 (−0.72, 7.93)	0.0946
Public expenditure on health (% of GDP)	5.43 (1.62, 9.25)	0.007	0.18 (−25.20, 25.57)	0.9876
Psychiatric hospital beds per 100 000 inhabitants	−0.04 (−0.22, 0.13)	0.626	−2.22 (−4.34, −0.11)	0.0411
Phase (end, vs start [reference])	22.64 (12.02, 33.26)	<0.001	–	–
R^2^	0.548		0.429	

Table 3 shows the age disparity in receiving antidepressants. Compared with people aged 50–64, people aged 65 or over had a lower likelihood of receiving antidepressants in 15 of 19 countries, with the smallest differential reported in Luxembourg (AOR 0.70 [0.49, 0.98]) and the highest in Germany (AOR 0.28 [0.21, 0.37]). This disparity widened further in 12 of 15 countries from 2011–2015 to 2015–2018 (AORs for trend < 1, *p* < 0.05). Unlike other European countries, people aged 65 or over in the UK had a 35% higher likelihood of receiving antidepressants compared to 50–64 year olds (AOR 1.35 [1.05, 1.73]). Data on younger people were only available in the UK, in which younger people (especially those aged 13–19) were less likely to receive antidepressants during 2014–2018. They were 94% less likely to receive antidepressants than the reference group (AOR 0.06 [0.03, 0.12]), with no change in this gap from 2014 to 2018 (AOR for trend 3.74 [0.71, 19.69]). In contrast, we detected no age disparity in Israel, Netherlands, or Portugal.

**Table 3:** Percentage receiving antidepressants (among those screening positive for depression) by phase and age.

Country	Age	Start phase	End phase	Adjusted OR (95%CI)	Adjusted OR for trend (95%CI)
Austria	50–64	41.58 (35.2, 48.26)	96.02 (92.04, 98.05)	Reference	Reference
	65+	24.99 (21.31, 29.06)	67.92 (61.33, 73.86)	0.33 (0.24, 0.44)***	0.19 (0.08, 0.45)***
Belgium	50–64	33.4 (29.39, 37.68)	86.2 (81.89, 89.61)	Reference	Reference
	65+	28.57 (25.69, 31.63)	51.01 (46.9, 55.1)	0.42 (0.35, 0.52)***	0.21 (0.14, 0.33)***
Czech Republic	50–64	27.25 (19.69, 36.39)	88.89 (79.3, 94.35)	Reference	Reference
	65+	20.89 (17.77, 24.39)	61.7 (54.92, 68.05)	0.45 (0.31, 0.66)***	0.34 (0.15, 0.79)*
Denmark	50–64	27.98 (23.89, 32.46)	66.36 (57.63, 74.1)	Reference	Reference
	65+	32.75 (28.17, 37.69)	37.57 (31.26, 44.34)	0.64 (0.49, 0.84)**	0.24 (0.14, 0.41)***
Estonia	50–64	15.14 (12.38, 18.38)	16.17 (13.28, 19.55)	Reference	Reference
	65+	10.41 (8.86, 12.19)	11.54 (9.81, 13.53)	0.55 (0.44, 0.69)***	1.06 (0.70, 1.62)
France	50–64	25.39 (21.76, 29.41)	76.33 (69.28, 82.18)	Reference	Reference
	65+	24.12 (21.45, 27.01)	43.65 (39.08, 48.34)	0.51 (0.41, 0.64)***	0.26 (0.16, 0.42)***
Germany	50–64	20.93 (18.08, 24.11)	87.7 (81.62, 91.97)	Reference	Reference
	65+	13.73 (11.11, 16.86)	40.6 (33.81, 47.77)	0.28 (0.21, 0.37)***	0.17 (0.09, 0.31)***
Greece	50–64	18.85 (15.71, 22.45)	52.69 (39.88, 65.15)	Reference	Reference
	65+	18.82 (16.34, 21.58)	33.26 (28.9, 37.94)	0.64 (0.48, 0.85)**	0.46 (0.25, 0.87)*
Israel	50–64	27.87 (16.2, 43.58)	29.93 (14.56, 51.72)	Reference	Reference
	65+	36.01 (30.56, 41.85)	20.66 (16.64, 25.36)	0.82 (0.45, 1.49)	0.51 (0.18, 1.42)
Italy	50–64	17.29 (13.59, 21.73)	71.13 (61.64, 79.06)	Reference	Reference
	65+	17.67 (15.27, 20.35)	33.08 (29.11, 37.32)	0.51 (0.38, 0.68)***	0.19 (0.11, 0.34)***
Luxembourg	50–64	25.84 (20.72, 31.72)	26.53 (19.84, 34.5)	Reference	Reference
	65+	27.67 (21.66, 34.61)	19.6 (14.67, 25.69)	0.70 (0.49, 0.98)*	0.61 (0.32, 1.20)
Netherlands	50–64	25.52 (17.23, 36.08)	24.09 (20.07, 28.63)	Reference	Reference
	65+	20.46 (16.53, 25.05)	20.91 (17.58, 24.68)	0.71 (0.41, 1.23)	1.08 (0.59, 1.97)
Poland	50–64	15.44 (10.67, 21.82)	70.95 (63.76, 77.22)	Reference	Reference
	65+	18.98 (15.23, 23.41)	37.56 (32.98, 42.37)	0.59 (0.41, 0.83)**	0.20 (0.11, 0.36)***
Portugal	50–64	34.4 (24.52, 45.84)	45.12 (32.58, 58.3)	Reference	Reference
	65+	24.51 (16.8, 34.29)	43.29 (32.9, 54.31)	0.68 (0.41, 1.12)	1.57 (0.61, 3.97)
Slovenia	50–64	15.35 (10.2, 22.45)	22.27 (17.65, 27.7)	Reference	Reference
	65+	10.92 (8.17, 14.44)	18.73 (15.67, 22.22)	0.63 (0.44, 0.89)**	1.11 (0.58, 2.12)
Spain	50–64	37.67 (30.04, 45.97)	87.16 (79.46, 92.25)	Reference	Reference
	65+	33.97 (30.33, 37.8)	48.53 (43.95, 53.13)	0.42 (0.31, 0.58)***	0.17 (0.08, 0.34)***
Sweden	50–64	30.85 (25.64, 36.59)	93.24 (86.32, 96.79)	Reference	Reference
	65+	26.7 (23, 30.76)	47.92 (41.99, 53.91)	0.39 (0.29, 0.53)***	0.08 (0.04, 0.20)***
Switzerland	50–64	28.25 (22.72, 34.52)	78.89 (67.69, 86.95)	Reference	Reference
	65+	22.52 (18.29, 27.4)	46.48 (38.95, 54.17)	0.47 (0.34, 0.66)***	0.32 (0.15, 0.67)**
UK	13–19	1.93 (0.47, 7.62)	6.65 (3.09, 13.71)	0.06 (0.03, 0.12)***	3.74 (0.71, 19.69)
	20–24	14.81 (7.43, 27.34)	17.99 (11.1, 27.82)	0.23 (0.14, 0.38)***	2.05 (0.69, 6.05)
	25–49	32.25 (28.42, 36.34)	28.13 (24.42, 32.17)	0.53 (0.43, 0.64)***	1.02 (0.68, 1.54)
	50–64	47.82 (42.77, 52.9)	42.71 (38, 47.55)	Reference	Reference
	65+	50.8 (45.53, 56.04)	44.13 (39.31, 49.07)	1.35 (1.05, 1.73)*	0.94 (0.61, 1.46)

Adjusted odds ratios (ORs) and their 95% confidence intervals (CIs) were estimated from weighted logistic regression, with antidepressant receipt as the dependent variable and age as the predictor, controlling for sex, phase, wealth status, and physical disability. Adjusted ORs for trends (with 95%CIs) were estimated by adding an age × phase (start [reference] vs end) term into the previous model and estimating from the interaction coefficient. *** *p* < 0.001; ** *p* < 0.01; * *p* < *p* < 0.05; . *p* < 0.1.

**Figure 2:**
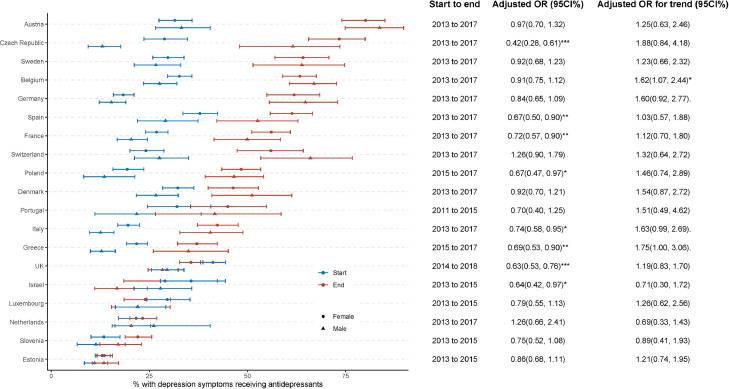
**Percentage receiving antidepressants (among those screening positive for depression) by phase and sex.** Adjusted odds ratios (ORs) and their 95% confidence intervals (CIs) were estimated from weighted logistic regression, with antidepressant receipt as the dependent variable and sex (female [reference] vs male) as the predictor, controlling for age, phase, wealth status, and physical disability. Adjusted ORs for trends (with 95%CIs) were estimated by adding a sex × phase (start [reference] vs end) term into the previous model and estimating from the interaction coefficient. *** *p* < 0.001; ** *p* < 0.01; * *p* < 0.05; . *p* < 0.1.

Figure 2 shows the sex disparity in receipt of antidepressants. Men were less likely to receive antidepressants in 8 out of 19 countries, with the smallest gap being in Italy (AOR 0.74 [0.58, 0.95]) and the highest in the Czech Republic (AOR 0.42 [0.28, 0.61]). We detected no significant change in these disparities from 2011–2015 to 2015–2018 (AOR for trend, *p* > 0.05). In Belgium, although there was no overall sex disparity in receiving antidepressants (AOR 0.91 [0.75, 1.12]), over time, men became more likely to receive antidepressants (AOR for trend 1.62 [1.07, 2.44]).

Table 4 shows the relationship between wealth status and antidepressant receipt (amongst those screening positive for depressive symptoms). In more than half of the countries, there was no wealth status effect, but there were some such effects in Austria, Belgium, Czech Republic, Denmark, Luxembourg, Slovenia, Spain, and Sweden. In Austria, Belgium, Denmark, Slovenia, Spain, and Sweden, some groups of more affluent people were less likely to receive antidepressants than those of the lowest wealth status. In Belgium, this wealth status disparity narrowed from 2013 to 2017, with richest people becoming more likely to receive antidepressants (AOR for trend 2.14 [1.15, 3.97]). In Sweden, this wealth status disparity also narrowed from 2013 to 2017, with middle-to-high-income people (AOR for trend 2.39 [1.01, 5.64]) and highest-income people (AOR for trend 3.03 [1.22, 7.54]) becoming more likely to receive antidepressants. In the Czech Republic and Luxembourg, some groups of higher-income people were more likely to receive antidepressants than those of the lowest wealth status (AORs > 1, *p* < 0.05), with no change in this wealth status disparity over time (AOR for trend, *p* > 0.05).

**Table 4:** Percentage receiving antidepressants (among those screening positive for depression) by phase and wealth status.

Country	Wealth status (quintile)	Start phase	End phase	Adjusted OR (95%CI)	Adjusted OR for trend (95%CI)
Austria	1 (low)	37.48 (30.43, 45.1)	82.8 (73.58, 89.27)	Reference	Reference
	2	25.62 (19.08, 33.47)	69.27 (55.92, 80.02)	0.58 (0.37, 0.89)*	1.02 (0.42, 2.46)
	3	27.82 (20.87, 36.03)	84.53 (74.74, 90.98)	0.83 (0.54, 1.25)	1.77 (0.73, 4.26)
	4	29.35 (21.92, 38.07)	87.77 (75.98, 94.21)	0.82 (0.52, 1.27)	2.44 (0.88, 6.69).
	5 (high)	39.24 (29.75, 49.62)	78.08 (60.85, 89.09)	1.04 (0.65, 1.68)	0.96 (0.34, 2.75)
Belgium	1 (low)	36.25 (31.34, 41.46)	65.49 (58.99, 71.45)	Reference	Reference
	2	29.07 (24.24, 34.42)	62.58 (54.2, 70.28)	0.77 (0.59, 1.01).	1.15 (0.68, 1.95)
	3	30.45 (25.23, 36.23)	62.74 (53.77, 70.91)	0.79 (0.60, 1.06)	1.13 (0.64, 1.99)
	4	31.51 (25.63, 38.04)	62.8 (54.51, 70.4)	0.79 (0.59, 1.05)	1.16 (0.66, 2.05)
	5 (high)	25.36 (19.62, 32.11)	69.46 (60.52, 77.14)	0.70 (0.51, 0.94)*	2.14 (1.15, 3.97)*
Czech Republic	1 (low)	16.99 (11.18, 24.97)	60.35 (41.13, 76.83)	Reference	Reference
	2	22.98 (18.6, 28.04)	70.96 (61.92, 78.6)	1.52 (0.96, 2.44).	0.94 (0.35, 2.56)
	3	30.99 (18.91, 46.38)	62.51 (50.6, 73.08)	1.84 (0.98, 3.46).	0.54 (0.17, 1.75)
	4	28.68 (18.76, 41.18)	79.39 (57.64, 91.6)	1.86 (1.05, 3.25)*	0.84 (0.23, 3.00)
	5 (high)	18.74 (12.62, 26.89)	74.99 (58.88, 86.26)	1.34 (0.79, 2.27)	1.40 (0.42, 4.71)
Denmark	1 (low)	34.94 (28.8, 41.63)	56.77 (46.38, 66.6)	Reference	Reference
	2	32.21 (25.52, 39.71)	41.84 (30.21, 54.45)	0.71 (0.49, 1.02).	0.61 (0.28, 1.32)
	3	28.61 (22.24, 35.97)	38.02 (27.67, 49.58)	0.57 (0.39, 0.82)**	0.63 (0.30, 1.32)
	4	26.66 (20.25, 34.22)	60.7 (45.48, 74.09)	0.71 (0.49, 1.04).	1.80 (0.77, 4.18)
	5 (high)	22.68 (15.65, 31.7)	40.5 (27.19, 55.38)	0.45 (0.29, 0.70)***	0.96 (0.39, 2.32)
Estonia	1 (low)	14.18 (10.42, 19)	16.11 (12.16, 21.02)	Reference	Reference
	2	10.87 (8.61, 13.65)	13.35 (10.62, 16.65)	0.87 (0.64, 1.20)	1.14 (0.62, 2.08)
	3	10.07 (7.23, 13.85)	13.35 (10.12, 17.41)	0.85 (0.60, 1.20)	1.19 (0.60, 2.34)
	4	14.91 (10.66, 20.47)	11.23 (7.9, 15.73)	0.88 (0.61, 1.27)	0.61 (0.30, 1.26)
	5 (high)	15.32 (11.54, 20.05)	12.56 (8.86, 17.52)	1.03 (0.73, 1.46)	0.70 (0.35, 1.42)
France	1 (low)	24.74 (20.36, 29.71)	55.3 (47.22, 63.11)	Reference	Reference
	2	23.05 (18.89, 27.8)	64.12 (54.38, 72.82)	1.09 (0.82, 1.45)	1.54 (0.83, 2.86)
	3	25.34 (20.4, 31.01)	54.85 (45.14, 64.2)	1.03 (0.76, 1.40)	0.95 (0.51, 1.77)
	4	25.64 (20.14, 32.04)	45.75 (35.28, 56.62)	0.88 (0.63, 1.22)	0.68 (0.35, 1.31)
	5 (high)	25.5 (20.11, 31.75)	50.73 (40.02, 61.37)	0.98 (0.70, 1.35)	0.78 (0.40, 1.51)
Germany	1 (low)	17.92 (13.99, 22.66)	53.99 (43.3, 64.32)	Reference	Reference
	2	17.7 (13.75, 22.48)	66.2 (54.24, 76.4)	1.21 (0.83, 1.75)	1.46 (0.68, 3.16)
	3	16.16 (11.85, 21.64)	64.5 (49.19, 77.32)	1.11 (0.74, 1.65)	1.77 (0.74, 4.18)
	4	17.22 (12.96, 22.51)	70.87 (58.81, 80.56)	1.26 (0.87, 1.84)	1.99 (0.92, 4.31).
	5 (high)	16.91 (12.62, 22.29)	67.91 (53.75, 79.4)	1.15 (0.78, 1.72)	1.90 (0.81, 4.48)
Greece	1 (low)	18.25 (14.39, 22.88)	33.9 (24.62, 44.63)	Reference	Reference
	2	22.25 (17.82, 27.4)	39.29 (31.95, 47.15)	1.28 (0.92, 1.77)	0.94 (0.48, 1.86)
	3	19.46 (15.08, 24.74)	33.43 (24.57, 43.64)	1.11 (0.77, 1.58)	0.90 (0.43, 1.93)
	4	15.39 (11.39, 20.46)	36.96 (25.73, 49.81)	0.94 (0.64, 1.38)	1.45 (0.64, 3.32)
	5 (high)	18 (13.76, 23.2)	36.55 (23.58, 51.82)	1.05 (0.71, 1.54)	1.20 (0.50, 2.92)
Israel	1 (low)	32.79 (18.72, 50.82)	18.62 (12.38, 27.04)	Reference	Reference
	2	29.65 (20.8, 40.35)	40.04 (18.14, 66.82)	1.60 (0.74, 3.46)	3.25 (0.90, 11.70).
	3	40.57 (30.09, 52)	23.76 (14.28, 36.85)	1.60 (0.90, 2.80)	1.07 (0.35, 3.22)
	4	26.96 (17.02, 39.93)	19.28 (11.05, 31.47)	0.90 (0.47, 1.73)	1.39 (0.40, 4.85)
	5 (high)	37.48 (25.21, 51.6)	15.15 (7.9, 27.11)	1.25 (0.66, 2.32)	0.52 (0.15, 1.80)
Italy	1 (low)	15.6 (12.01, 20.02)	40.06 (32.19, 48.47)	Reference	Reference
	2	18.25 (13.92, 23.57)	41.07 (32.37, 50.37)	1.19 (0.86, 1.65)	1.00 (0.52, 1.92)
	3	20.56 (15.77, 26.35)	34.41 (26.3, 43.56)	1.15 (0.81, 1.62)	0.59 (0.31, 1.16)
	4	15.52 (11.35, 20.86)	46.17 (33.19, 59.69)	1.06 (0.73, 1.55)	1.46 (0.67, 3.19)
	5 (high)	18.04 (13.22, 24.12)	52.03 (41.72, 62.16)	1.34 (0.94, 1.90)	1.55 (0.76, 3.13)
Luxembourg	1 (low)	21.87 (14.77, 31.13)	18.14 (11.97, 26.53)	Reference	Reference
	2	24.49 (17.32, 33.42)	28.41 (18.63, 40.74)	1.48 (0.90, 2.44)	1.60 (0.61, 4.22)
	3	33.83 (24.29, 44.9)	28.27 (18.83, 40.12)	1.86 (1.14, 3.03)*	0.99 (0.37, 2.64)
	4	20.62 (12.51, 32.06)	30.74 (18.79, 45.98)	1.52 (0.84, 2.75)	2.12 (0.71, 6.30)
	5 (high)	35.33 (24.57, 47.82)	12.34 (6.07, 23.45)	1.21 (0.70, 2.05)	0.32 (0.10, 1.00).
Netherlands	1 (low)	24.18 (15.83, 35.1)	22.6 (17.38, 28.83)	Reference	Reference
	2	26.98 (14.38, 44.84)	20 (14.93, 26.26)	1.08 (0.46, 2.53)	0.82 (0.31, 2.14)
	3	17.2 (11.09, 25.72)	25 (19.11, 31.99)	0.68 (0.32, 1.43)	1.68 (0.69, 4.10)
	4	24.03 (17.41, 32.17)	22.62 (16.87, 29.63)	1.01 (0.50, 2.01)	1.02 (0.44, 2.39)
	5 (high)	18.53 (12.22, 27.08)	21.23 (15.29, 28.71)	0.74 (0.36, 1.52)	1.35 (0.56, 3.25)
Poland	1 (low)	20.66 (12.29, 32.61)	45.12 (34.87, 55.8)	Reference	Reference
	2	15.41 (11.01, 21.15)	46.61 (39.18, 54.2)	0.79 (0.48, 1.31)	1.48 (0.61, 3.56)
	3	17.91 (11.28, 27.25)	45.42 (36.5, 54.65)	0.93 (0.53, 1.65)	1.16 (0.44, 3.10)
	4	13.69 (8.12, 22.17)	48.51 (38.55, 58.58)	0.80 (0.46, 1.39)	1.88 (0.68, 5.21)
	5 (high)	18.74 (12.2, 27.68)	56.59 (46.19, 66.45)	1.07 (0.61, 1.90)	1.67 (0.63, 4.35)
Portugal	1 (low)	22.44 (14.32, 33.35)	42.22 (24.59, 62.1)	Reference	Reference
	2	26.99 (14.72, 44.19)	29.11 (18.35, 42.87)	0.78 (0.38, 1.60)	0.47 (0.12, 1.82)
	3	21.7 (11.21, 37.84)	50.69 (34.97, 66.28)	1.23 (0.61, 2.51)	1.38 (0.36, 5.37)
	4	33.61 (19.08, 52.09)	46.17 (30.25, 62.9)	1.57 (0.75, 3.29)	0.77 (0.19, 3.06)
	5 (high)	37.93 (23.02, 55.53)	56.69 (35.66, 75.56)	2.05 (0.96, 4.44).	0.84 (0.20, 3.56)
Slovenia	1 (low)	19.7 (10.92, 32.94)	24.28 (18.17, 31.66)	Reference	Reference
	2	10.9 (7.36, 15.86)	20.58 (15.38, 26.99)	0.67 (0.42, 1.06).	1.51 (0.59, 3.82)
	3	9.3 (5.31, 15.77)	17.23 (11.83, 24.4)	0.57 (0.34, 0.94)*	1.58 (0.54, 4.57)
	4	9.62 (5.65, 15.91)	22.96 (16.17, 31.53)	0.68 (0.41, 1.13)	2.10 (0.74, 5.93)
	5 (high)	15.13 (8.59, 25.25)	16.97 (12.11, 23.26)	0.68 (0.41, 1.14)	0.84 (0.29, 2.41)
Spain	1 (low)	38.18 (30.18, 46.89)	56.05 (46.44, 65.23)	Reference	Reference
	2	29.98 (23.78, 37)	51.07 (42.22, 59.86)	0.67 (0.46, 0.97)*	1.22 (0.60, 2.46)
	3	38.06 (29.59, 47.32)	67.9 (57.54, 76.76)	1.09 (0.73, 1.63)	1.67 (0.78, 3.53)
	4	38.44 (28.23, 49.77)	49.76 (38.79, 60.76)	0.86 (0.54, 1.36)	0.83 (0.35, 1.93)
	5 (high)	34.42 (26.44, 43.39)	69.84 (57.55, 79.82)	1.06 (0.70, 1.60)	1.95 (0.88, 4.35)
Sweden	1 (low)	34.16 (27.89, 41.04)	63.27 (52.02, 73.23)	Reference	Reference
	2	25.65 (19.37, 33.13)	57.51 (43.88, 70.08)	0.73 (0.50, 1.08)	1.45 (0.66, 3.19)
	3	28.23 (21.84, 35.65)	65.08 (49.6, 77.92)	0.82 (0.55, 1.21)	1.52 (0.66, 3.49)
	4	23.17 (16.06, 32.22)	65.39 (50.1, 78.04)	0.61 (0.40, 0.93)*	2.39 (1.01, 5.64)*
	5 (high)	26.6 (19.47, 35.19)	73.53 (57.73, 84.96)	0.67 (0.44, 1.03).	3.03 (1.22, 7.54)*
Switzerland	1 (low)	26.68 (19.38, 35.51)	48.81 (34.61, 63.21)	Reference	Reference
	2	23.58 (16.77, 32.08)	66.69 (49.13, 80.59)	1.15 (0.70, 1.88)	2.12 (0.74, 6.05)
	3	25.29 (18.14, 34.09)	61.73 (44.23, 76.63)	1.09 (0.66, 1.82)	1.46 (0.50, 4.26)
	4	28.64 (20.14, 38.98)	53.94 (37.2, 69.83)	1.12 (0.66, 1.88)	1.07 (0.38, 3.03)
	5 (high)	22.09 (14.65, 31.89)	66.55 (51.38, 78.93)	1.04 (0.63, 1.73)	2.29 (0.81, 6.42)
UK	1 (low)	35.66 (30.46, 41.23)	32.41 (27.59, 37.63)	Reference	Reference
	2	44.24 (37.48, 51.22)	32.53 (27.88, 37.56)	1.20 (0.93, 1.52)	0.75 (0.46, 1.23)
	3	39.28 (32.94, 46.01)	31.34 (26.31, 36.85)	0.98 (0.76, 1.26)	0.81 (0.49, 1.35)
	4	37.64 (31.13, 44.63)	37.12 (31.62, 42.98)	1.16 (0.90, 1.51)	1.02 (0.61, 1.72)
	5 (high)	34.51 (28.18, 41.45)	30.01 (24.45, 36.22)	0.90 (0.68, 1.17)	0.84 (0.49, 1.45)

Adjusted odds ratios (ORs) and their 95% confidence intervals (CIs) were estimated from weighted logistic regression, with antidepressant receipt as the dependent variable and wealth status as the predictor, controlling for age, sex, phase, and physical disability. Adjusted ORs for trends (with 95%CIs) were estimated by adding a wealth status × phase (start [reference] vs end) term into the previous model and estimating from the interaction coefficient. *** *p* < 0.001; ** *p* < 0.01; * *p* < 0.05; . *p* < 0.1.

**Figure 3:**
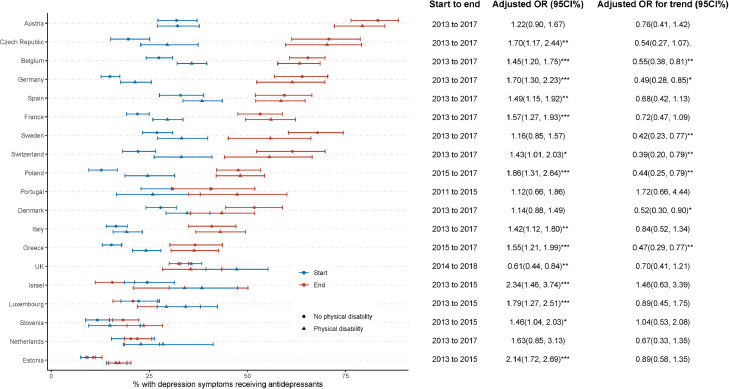
**Percentage receiving antidepressants (among those screening positive for depression) by phase and disability.** Adjusted odds ratios (ORs) and their 95% confidence intervals (CIs) were estimated from weighted logistic regression, with antidepressant receipt as the dependent variable and physical disability (versus “no physical disability” as the reference) as the predictor, controlling for age, sex, phase, and wealth status. Adjusted ORs for trends (with 95%CIs) were estimated by adding a physical disability × phase (start [reference] vs end) term into the previous model and estimating from the interaction coefficient. *** *p* < 0.001; ** *p* < 0.01; * *p* < 0.05; . *p* < 0.1.

Figure 3 shows the disparity in receiving antidepressants with respect to physical disability. In 13 of 19 countries, people with physical disability were more likely to receive antidepressants compared to those with no such disability. The smallest gap was in Italy (AOR 1.42 [1.12, 1.80]) and the largest gap was in Israel (AOR 2.34 [1.46, 3.74]). This disability disparity narrowed in 5 of 13 countries from 2011–2015 to 2015–2018 (AORs for trend < 1, *p* < 0.05). In the UK, after adjustment for age, phase, wealth status, and physical disability, in contrast to the unadjusted risk, people having physical disability were less likely to receive antidepressants than those without such disability (AOR 0.61 [0.44, 0.84]), with no change in the size of the gap from 2014 to 2018.

## Discussion

### Statement of principal findings

Usage of antidepressants by those who screened positive for depressive symptoms increased greatly from 2011–2015 to 2015–2018, but the magnitude of change varied widely among the European countries studied. The percentage receiving antidepressants was positively associated with the health expenditure in a country, but not with affordability (reflected by gross national income per capita) or availability of specific measured healthcare resources (the number of psychiatrists, general practitioners, or psychiatric hospitals beds). Increased usage of antidepressants was associated with a decrease in psychiatric bed provision, but not with changes in the other four country-level factors. Salient subgroup disparities were detected for sex, age, and physical disability. From the first phase (2011–2015) to the second (2015–2018), the age disparity widened, the sex disparity persisted, and the physical disability disparity narrowed. Disparity by wealth status was relatively weak.

### Possible explanations and comparison with other studies

Increases in usage of antidepressants by those with depressive symptoms may reflect improvements in access, via a concerted effort by European countries to integrate mental health in primary care, including de-institutionalization and developing community-based care.^31^^,^^32^ Our findings are consistent with previous European studies on antidepressant use within long-term care facilities, and on hospitalization rates for mental disorders.^4^^,^^5^^,^^33^, ^34^, ^35^ Our finding of the negative association between the change in the proportion with depressive symptoms receiving antidepressants and reductions in psychiatric hospital beds is also consistent with the influence of de-institutionalization. However, the changes observed were not consistently in line with the process of community-based care in the countries studied. For instance, results varied between countries considered to have well established and strong community-oriented delivery systems, such as the UK, Italy, Spain, Austria, and France.^32^ Our country-level analyses (Table 2) provide more detail on this variation, showing that the percentage receiving antidepressants was associated with overall health expenditure in a country, but not with GNI or specific mental health care resource measures (general practitioners, psychiatrists, psychiatric beds). Furthermore, the progress made by countries was negatively associated with the changes in psychiatric beds, but not other country-level measures. Possible explanations include that financed public campaigns to inform the population about depression and to educate frontline professionals may have reduced stigma and encouraged people to seek help for depression.^4^^,^^31^ Compared to additional financial input, reconfiguration of existing services could also have increased access to, or use of, antidepressants. In addition, country-specific actions, such as clinical guidelines, may also affect prescription practice.^34^, ^35^, ^36^ For instance, the UK Improving Access to Psychological Therapies (IAPT) programme emphasizes psychological treatment (i.e. a non-pharmacological approach),^37^ and could explain why the percentage receiving antidepressants (among those screening positive for depressive symptoms) decreased in the UK.

The results for 2015–2018 (Figure 1) also indicate variable usage of antidepressants, ranging from Austria (approximately 20%) to Estonia (more than 85%), compared to the mean of 38.7%. Patient preference, local clinical practice, under-accessibility, and potentially over-prescription of antidepressants might all contribute,^5^^,^^38^ as might differences in national policies or interventions. In addition, there is evidence that promotion by the pharmaceutical industry is positively associated with antidepressant prescription.^39^ Variations in this effect (which might be affected by a variety of factors such as limitations on promotional activities, promotional budgets, type of relationships with prescribers, and professional training) might explain some part of the cross-national differences in temporal trends; however, we did not have data enabling us to measure any such effect in our study. Systematic study of these variations in practice, including economic evaluations, could enhance practice and clinical outcomes in Europe and beyond.

The high rates of antidepressant use and large increases in some countries studied are comparable to the USA (69.4% in 2015, increased from 52.1% in 1996),^40^ but there are reasons for caution. People included in the present study had screened positive for depressive symptoms but that does not necessarily reflect a clinical diagnosis of a depressive disorder (self-report scales are imprecise with respect to formal diagnosis^18^), or its severity if present. Use of antidepressants may have been inappropriate for those screening positive but without the disorder. Those who did have depression might have been treated appropriately with psychological therapy alone, declined antidepressants, or stopped antidepressant treatment following improvement or because of side effects. Therefore, high prevalence, or increases in, antidepressant usage (and thus prescription) in a given country does not necessarily imply better management. Potential alternative reasons for this trend include over-prescription and a use of antidepressants instead of an appropriate non-pharmacological therapy.

People aged 65 or over were less likely to be prescribed antidepressants than people aged 50–64 in 15 of 19 European countries studied, a finding consistent with previous studies showing a decrease in antidepressant usage with age, or highest usage in middle-aged populations.^34^^,^^35^^,^^41^ Older populations are more likely to present with multiple diseases resulting in polypharmacy, and are more likely to suffer cognitive and functional impairment. Physicians may, as a result, be reluctant to prescribe antidepressant medications to avoid potential adverse drug–drug reactions, or may prefer psychological therapies because of the suboptimal effectiveness of antidepressant medications among frail individuals with cognitive and functional impairment^42^, ^43^, ^44^; patient preference in considering of benefits and adverse effects of antidepressant use versus psychological therapies may also contribute.^45^^,^^46^ Alternatively, these findings could also indicate inappropriate under-prescription of antidepressants to the older population, as suggested by findings from Germany (using data from 2008 to 2010) and the United States (using data from 2004 to 2005).^47^^,^^48^ An updated study is needed, as our findings suggest that the age disparity in access to antidepressants has widened from 2011–2015 to 2015–2018—particularly as the underuse of antidepressants for depressive disorders is associated with increased disability, worsening of clinical outcomes and increased mortality.^49^ A further reason for the increase in age disparity might be population/sample aging (e.g. if the average age of people in the ≥65-year-old group was higher during 2015–2018 than 2011–2015). In contrast to other European countries, people aged 65 or over in the UK were more likely to be prescribed antidepressants than people aged 50–64. Prior work has also suggested over-prescription of antidepressants for older people, identified in England and Wales using data from 1993 to 1997.^50^

Some large-scale studies have found that the peak onset for depression is from the late teens to about 20 years old^51^ (though estimates have varied^52^). We found that UK people aged 13–19 were 94% less likely to receive antidepressants, followed by people aged 20–24 (77%), than people aged 50–64. Given evidence from data gathered at a similar time of increasing prevalence of depression among young people in the UK, we should be concerned about under-prescription.^53^^,^^54^ However, UK clinical guidelines advise psychological therapies as the first-line treatment, unless depression is severe, for those under 18 years old.^55^ There is a great deal of media and policy attention to mental health in young people, a mental health workforce shortage, and consequent referral pressures impeding access to child and adolescent mental health services.^56^ We lacked data on younger age groups from other European countries, but access to child and adolescent mental health services is also sometimes suboptimal in other European countries.^57^ More attention to depression in young people is needed, given the particularly high developmental price of impairment during this key life stage, with further evidence of worse outcomes in recent cohorts.^58^

Subgroup disparities were also identified in relation to sex and physical disability. In accordance with previous studies,^4^^,^^5^^,^^33^^,^^59^^,^^60^ we found that males were significantly less likely to receive antidepressants in 8 of 19 countries. Physicians’ prescribing behaviour may be influenced by sex difference in external expression of emotions, or because men may be less likely to seek help.^33^^,^^59^^,^^60^ This disparity changed little from 2011–2015 to 2015–2018, even widening in Belgium. Our finding that people with physical disability were significantly more likely to receive antidepressants in 13 of 19 countries is also consistent with previous studies.^59^^,^^61^ Physical disability is associated with a higher prevalence of depression, so it is possible that depression is more likely to be recognized and treated in this context.^59^^,^^61^ This disability disparity narrowed in 5 of 13 countries primarily because of the improvement for people without physical disability, including Belgium, Germany, Switzerland, Poland, and Greece, which yet might suggest a degree of diagnostic overshadowing or lack of access to treatment for people with disability. The disparity in the UK was different from other European countries, in that people with physical disability in the UK (and screening positive for depressive symptoms) were less likely to receive antidepressants than those having no such disability, a picture that did not change from 2014 to 2018. A further study is needed to explore if this results from the use of psychological therapies or under-treatment, or potentially methodological differences between HSE and SHARE (discussed below).

Significant disparity by wealth status was found in 8/19 countries studied, with variation in the direction of the relationship between individual wealth and antidepressant. This finding is to some extent in line with previous studies; for instance, a study from Peru concluded that^62^ individuals with lower levels of wealth were less likely to be treated for depression,^62^ while a study from Denmark^63^ indicated that having higher income was associated with lower odds of using antidepressants.^63^ Our findings are likely to reflect the complex factors influencing the desirability of medication or psychological therapy as well as access that individual wealth could buy.

### Strengths and limitations

To our knowledge, this is the first study to assess the accessibility of antidepressants for those who screen positive for depressive symptoms following the end of the EMHAP (2013–2020). The repetitive cross-sectional representative data enabled the exploration of the progress made towards this goal. Subgroup analysis allowed a more nuanced and practical assessment of progress before the COVID-19 pandemic. The SHARE study used standardized methodology across its participating countries explicitly to support cross-country comparison, and we used this alongside measures of national health system factors to compare countries’ responses.

However, our findings should be treated with caution in that absolute comparisons between the UK and other countries should not be made, and comparisons between countries are subject to some caveats. The two surveys in this study (HSE, SHARE) have differences including: (a) the age range covered; (b) the sampling methods and the sample frames; (c) the instrument used to collect the information on depression symptoms; (d) measurement of wealth status; and (e) the method of cataloguing antidepressants. The antidepressant recording methods may have had different biases: for example, HSE's method, based on formulary drug class, would categorize a tricyclic antidepressant prescribed for neuropathic pain in the antidepressant category, but would omit lamotrigine for bipolar depression, while SHARE's self-rating method, based on perceived purpose, would enable participants to include benzodiazepines in the anxiolytic/antidepressant category but might exclude antidepressant drugs prescribed for an indication other than depression. Within SHARE, sampling methods are designed to be as similar as possible, but are not identical. We used survey weighting to adjust for differences caused by the survey design to make the data representative for each country and each period, and also analysed the data for each country separately. While this method provides robust handling of individual countries (as a given country was surveyed consistently over time), and consistent methods were used across 18/19 countries, statistical comparisons between countries, particularly between the UK and other countries, should be viewed with care. For other country-specific reasons (discussed above/below), Figure 1 should not be taken as a measure of countries’ performance against some kind of standard.

Our study also has a number of other limitations. First, informal support, other measures within primary care (such as exercise and sleep management), and psychological therapies are important complements to antidepressants for treating depression.^31^^,^^64^, ^65^, ^66^, ^67^ However, no corresponding data from primary care were available. Second, there was a lack of clinical confirmation of diagnosis, as data were drawn from large-scale population surveys using self-administered instruments. Additionally, as a result, we were unable to distinguish unipolar depression (major depressive disorder) and bipolar depression; the latter is often not treated with conventional antidepressants. Third, the self-administered instruments have only been validated for binary detection of depressive disorders, and do not provide accurate quantification of severity. No data on antidepressant type/dose/duration were available. These limitations prevent us from establishing the relationship between degree of need and antidepressant prescription or measuring any potential over-prescription. Fourth, people with depression may have been successfully treated, and thus have been taking antidepressants but without residual symptoms to be identified by the survey instruments; such people would have been missed by this approach, underestimating the proportion of people with depression being treated with antidepressants. We note also the potential for bias in the other direction by including such people, given (for example) that monoaminergic antidepressants are also used for other conditions such as migraine or neuropathic pain syndromes. Fifth, the results of country-level analyses should be treated with caution. For instance, in most countries a considerable proportion of antidepressant prescribing is by non-psychiatrists; although we took into account the number of GPs, other types of non-psychiatric specialists, such as general physicians (internists) also make such prescriptions. Public expenditure on health is a common index to reflect country-level input and healthcare affordability, but in countries without universal health coverage, this measure may not account adequately for the requirement for patients to pay directly for antidepressant prescriptions, sums that are not included in national health expenditure evaluations. Psychiatric bed count and antidepressant receipt may be only loosely associated, because a majority of people with major depressive disorder are not treated in hospital psychiatric settings (though bed counts and the number of psychiatrists may be proxies for spending on secondary mental health care more broadly). Therefore, the national-level results are only a macroscopic reflection with multiple possible underlying reasons. For instance, spending saved by reducing psychiatric beds might be used to improve mental health care in primary (or outpatient secondary) care, or promote awareness in the general population. Sixth, since within-country analyses were of a priori interest, such comparisons were made without correction for multiple comparisons across all countries to reduce the chance of type II errors, though this of course increases the potential for type I error.

### Generalizability, implications, and conclusions

Usage of antidepressants by those who screen positive for depressive symptoms has increased greatly among European countries, but the wide variance and subgroup disparities raise the possibilities of both under-accessibility and over-prescription. There were disparities in antidepressant usage by age, sex, and physical disability. The difference in usage by age deserves particular attention, as this disparity has in some cases widened. Our findings suggest that characteristics other than clinical need influence access to antidepressants for those who screen positive for depressive symptoms, though there are limitations that may reduce generalizability to those with depressive disorder. Commissioners, practitioners, and policy makers could use these findings as one starting point to investigate and improve appropriate access to mental health treatments in their regions.

## Contributors

SC contributed to the concept and study design. SC conducted the analysis. SC, TJF, PBJ, and RNC interpreted the results. SC drafted the manuscript. TJF, PBJ, and RNC made critical revision of the manuscript for important intellectual content. All authors edited and approved the final manuscript.

### Data availability statement

The data are publicly available. The Health Survey for England can be accessed in UK Data Service (https://beta.ukdataservice.ac.uk/datacatalogue/series/series?id=2000021). The Survey of Health, Ageing and Retirement in Europe can be accessed here (http://www.share-project.org/data-access.html).

## Declaration of Interests

• SC declares no conflict of interest with this work.

• TF consults to Place2Be, a third sector organisation providing mental health support to schools.

• PBJ is a scientific advisory board member for MSD.

• RNC consults for Campden Instruments Ltd and receives royalties from Cambridge University Press, Cambridge Enterprise, and Routledge.

## Appendix Supplementary materials

Image, application 1

Image, application 2
