# Supplementary material for: Prevalence, progress, and subgroup disparities in pharmacological antidepressant treatment of those who screen positive for depressive symptoms: A repetitive cross-sectional study in 19 European countries
Source: Lancet Reg Health Eur. 2022 Mar 28;17:100368. doi: 10.1016/j.lanepe.2022.100368 (PMC8969158; doi:10.1016/j.lanepe.2022.100368)
Supplement: Supplementary file 2 [file mmc2.docx]

**Sub Fig 1. Flowchart of selection**
